# COVID-19 inflammatory signature in a Mozambican cohort: unchanged red blood series and reduced levels of IL-6 and other proinflammatory cytokines

**DOI:** 10.1186/s12879-024-10132-6

**Published:** 2024-11-11

**Authors:** Vânia Maphossa, Onélia Guiliche, Teresa Babetine, Celso Castiano, Osvaldo Inlamea, Marino Marengue, Igor Capitine, Lúcia Chambal, Almiro Tivane, Jahit Sacarlal, Eugênia Terra-Granado, Raquel Matavele Chissumba

**Affiliations:** 1 Instituto Nacional de Saúde, Maputo, Mozambique; 2 Faculdade de Medicina, Universidade Eduardo Mondlane, Maputo, Mozambique; 3 Hospital Geral do Polana Caniço, Maputo, Mozambique; 4 Hospital Central de Maputo, Maputo, Mozambique; 5 Centro de Pesquisas, Instituto Nacional de Câncer (INCA), Rio de Janeiro, Brazil; 6 Instituto Gonçalo Moniz, Fundação Oswaldo Cruz (FIOCRUZ), Bahia, Brazil; 7 Centro de Investigação e Desenvolvimento em Etnobotânica, Namaacha, Mozambique

**Keywords:** COVID-19, SARS-CoV-2, Biochemistry, Haematology, Cytokines, Immunological signature

## Abstract

**Background:**

Alterations in haematological, biochemical parameters and cytokine levels, were reported in patients with COVID-19, however, there is an underrepresentation of the African population, which could provide evidence for understanding SARS-CoV-2 pathogenesis and useful tools for clinical management of cases. In this study, we aimed to determine the haematological, biochemical and cytokine profile in Mozambican individuals with SARS-CoV-2.

**Methods:**

A cohort of 85 Mozambican individuals with RT-PCR SARS-CoV-2 results, was stratified into negative, asymptomatic, mild, moderate, and severe categories. Haematological, biochemical and cytokines measurement were performed on samples from the study participants. Principal component analysis (PCA) was performed to identify similar patterns among the study cases. Comparisons between groups were performed using the Kruskal-Wallis test. Receiver operating characteristic (ROC) and area under the curve (AUC) analysis were conducted to evaluate the ability of these parameters to distinguish severe from non-severe cases of SARS-CoV-2 infection.

**Results:**

SARS-CoV-2 infection was associated with a significant (*p* < 0.05) decrease in peripheral blood absolute counts of total lymphocytes and eosinophils, below the reference values along with no abnormal change (*p* > 0.05) in red blood cell count, haemoglobin, platelets and other red series parameters. At the serum level, SARS-CoV-2 infection was associated with an increase in serum levels of C-reactive protein (C-RP) and glucose above the reference values and to a significant reduction a significant (*p* < 0.05) reduction in levels of interferon-gamma (INF-γ), Tumour Necrosis Factor alfa (TNF-α) and the interleukin 1 beta (IL-1β) and IL-6 in severe cases, when compared to negative cases. Haematological, biochemical and cytokine profiles segregate severe from non-severe cases of COVID-19 with an excellent performance of C-RP (AUC = 0.95; *p* < 0.001) and good performance of lymphocytes (AUC = 0.88; *p* < 0.001) and IL-15 (AUC = 0.86; *p* < 0.001).

**Conclusion:**

The lack of variation in red and platelet series, coupled with a decrease in the levels of classical pro-inflammatory in severe cases, deviates from what has been reported in other contexts suggesting, that there may be peculiarities in COVID-19 manifestation within the context of this study population. Furthermore, these results identify parameters with potential for clinical management of COVID-19 and therefore good resource allocation, particularly for severe cases.

**Supplementary Information:**

The online version contains supplementary material available at 10.1186/s12879-024-10132-6.

## Background

Since the World Health Organization (WHO) declared COVID-19 a global pandemic, 774 593 066 confirmed cases of COVID-19 have been registered worldwide. Africa has been the least affected continent, accounting for 1% (957 575 cases) of the total cases and 2% (175 496 cases) of total deaths [1]. Since the outbreak, Mozambique has reported 233 731 confirmed cases and approximately 2 250 deaths [2]. The low numbers of cases and deaths observed in Africa are still under discussion and many theories associate this to epidemiologic prolife of the region and demographic factors such as age and genetics of the population [3–5]. The pathogenesis of COVID-19 is associated with the overproduction of cytokines, known as a cytokine storm [6] causing a hyperactivation of the immune system, dysregulation of body homeostasis, and dysfunction of several organs such as the lung (primary site of infection), kidneys, liver and heart [6]. Disease severity have been associated to an increase in cytokines such us: IL-1β, IL-6, INF-γ, TNF-α IL-10, IL-8, IL-18, P-10, MIP-1α, and MCP-1 [7, 8]. These findings led to cytokines being proposed not only as markers of prognosis but also as target for immunotherapies. In this regard, the effect of anti-IL-6 therapy for management of severe cases have been studied on clinical trials [8–10]. Alterations on serum parameters like C-reactive protein (C-RP) alanine aminotransferase (AST) and aspartate aminotransferase (ALT) [11] and haematological parameters such as lymphocytes and neutrophils, red blood cells and platelets counts have been associated to patients severity and survival [11–13]. Therefore, it is important to evaluate haematological and biochemical parameters, that are easily available but not fully explored on patient management. Furthermore, to date there is scarce information on signature of SARS-CoV-2 infection in the African context [14, 15] and a comprehensive approach to different paraments provide additional evidence for understanding the pathogenesis of SARS-CoV-2 and identification of tools for clinical management [16, 17]. In this study we evaluated haematological, biochemical, and cytokine level changes in Mozambican patients with different clinical presentations of SARS-CoV-2 infection.

## Methods

### Study population

A cross-sectional observational study was performed in participants aged more than 18 years recruited by convenience between February 2021 to January 2022. Individuals with negative RT-PCR for SARS-CoV-2, asymptomatic and mild cases were recruited from the SARS-CoV-2 Surveillance activities at Instituto Nacional de Saúde in Maputo and moderate and severe cases from the COVID-19 Isolation centers at Hospital Central de Maputo and Hospital Geral da Polana Caniço, both referral hospitals for the management of COVID- 19 cases in Maputo city.

### Data and sample collection

For each individual, clinical data were collected using pre-structured questionnaire with the following variables: demographic data, date of hospitalization, date of symptom evolution, HIV status, and the presence of comorbidities: diabetes mellitus, hypertension, cardiopathies, asthma, rhinitis, and sinusitis. Additionally, peripheral blood and a nasopharyngeal swab sample were collected upon admission to the hospital before the medication (for those hospitalized patients) see Additional file 1. Patients were grouped by clinical presentation according to the WHO [18] guidelines, as asymptomatic, mild, moderate and severe.

### HIV testing

HIV testing was performed for all participants according to the Mozambican national algorithm using the Determine HIV-1/2 test (Cat#7D2343SET) for screening, followed by the Uni-Gold (Cat# 1206502), for confirmation of positive results.

### SARS-CoV-2 testing

A nasopharyngeal swab was collected and placed in Viral Transport Medium (VTM) for further testing of SARS-CoV-2 via RT-PCR a Roche and (Cat# Cobas 6800) and GeneXpert (Cat# GXIV-4-D-10 C). The viral load was inferred through cycle threshold (CT) value.

### Haematological parameters quantification

Approximately 3 ml of EDTA blood was collected to perform complete cell blood count in 5 parts including neutrophils (NEUT), lymphocytes (LYMP), monocytes (MONO), eosinophils (EOS), basophils (BASO), at two Sysmex analysers (Cat# XS1000i and Cat# XN 550). After that NEUT, LYMP, MONO and platelets (PLT) were used to calculate in an Excel sheet the following ratio Lymphocyte to Monocyte ratio (LMR), Neutrophils to Lymphocyte Ratio (NLR) and Platelets to Lymphocyte Ratio (PLR).

### Biochemical parameters quantification

Whole blood in 5 ml serum-separating (SST) tube was centrifuge, and serum samples were used to quantify the biochemical parameters aspartate aminotransferase (AST), alanine aminotransferase (ALT), albumin (ALB), creatinine (CREA), glucose (GLUC), total Bilirubin (BILT), urea (URE), uric Acid (UA), cholesterol (CHOL), triglycerides (TRIG) and the inflammatory parameter C-reactive protein (C-RP) using a biochemical analyzer (Cat# COBAS C111).

### Cytokine quantification

Serum samples stored at -80^0^C were thawed and used for quantification of interferon gamma (INF-γ), tumour Necrosis Factor alfa (TNF-α) and the interleukin 1 beta (IL-1β), IL-2, IL-4, IL-5, IL-6, IL-7, IL-8, IL-15, IL-17, IL-18, IL-21,and IL-10 using the commercial Human Premixed Multi-Analyte kit (Cat# LXSAHM-14) and transform growth factor beta (TGF-β) using the commercial TGF-beta Premixed Magnetic Luminex kit (Cat# FCSTM17-1) according to the manufactures instructions. Readings were performed in the MAGPIX XPONET 4.3 analyser (Cat# MAGPIX-XPON42).

### Statistical analysis

The data were entered into Microsoft Excel sheets and analyzed using GraphPad Prism 9. Descriptive statistics were employed, medians and interquartile ranges were used to represent the distribution of sociodemographic characteristics among the clinical stages. The chi-square test was used to analyze the associations between categorical data. Principal component analysis (PCA) using separately haematological, biochemical, and cytokines datasets was performed to identify similar parameter patterns among the study cases. Comparisons between groups were assessed using Kruskal Wallis (post-hoc pairwise comparison by using Dunn test). Due to the low number of moderate cases (*n* = 5) these cases were not included in the between-groups analysis. Correlation was performed using the Spearman rank assuming the following interpretation of the r value, the strength of correlation as follows: 0-0.19 (very weak), 0.2–0.39 (weak), 0.40–0.59 (moderate), 0.6–0.79 (strong) and 0.8-1 (very strong). Receiver operating characteristic curve (ROC) and Area under the curve (AUC) analyses were also conducted to evaluate the ability of a particular parameter to distinguish severe from non-severe cases. The AUC values were interpreted as follows: 0.5–0.6 (failed), 0.6–0.7 (worthless), 0.7–0.8 (poor), 0.8–0.9 (good), and > 0.9 (excellent). All analyses were performed with a α = 0.05.

## Results

### Clinical and socio-demographic characteristics of patients

A total of 85 participants were enrolled in this study, 63 (74.1%) of whom had a positive RT-PCR for SARS-CoV-2. These participants with a positive rt-PCR were categorized as asymptomatic (*n* = 21; 24.7%), mild (*n* = 16; 18.8%), moderate (*n* = 5; 5.9%) and severe (*n* = 21; 24.7%) for COVID-19. Overall, 47 (53.3%) were female and 38 (44.7%) were male, with a median age of 39 years [IQR: 19–85 years]. However, compared with the other study participants, the moderate cases were older, with a median age of 58 years [IQR: 49.5–61 years]. Hypertension was the most common comorbidity (52.9%, 18/34) among the study participants, followed by diabetes (20,6%, 7/34), as shown in Table 1. The median duration of symptom onset were 5 days [IQR: 4-7.8 days], 4 [IQR: 3-5days] and 5 [IQR: 3–7 days] for those from mild, moderate, and severe cases, respectively. Dyspnea and vomiting were observed only in the severe cases (Additional file 2).

**Table 1:** Socio-demographic and clinical characteristics of the study population according to SARS-CoV-2 infection severity

Characteristics		Clinical state *n* (%)					Total on study population (%)	*P*- value*
		Negative	Positive (*n* = 63)					
			Asymptomatic	Mild	Moderate	Severe		
		*n* = 22 (25.9)	*n* = 21 (24.7)	*n* = 16 (18.8)	*n* = 5 (5.9)	*n* = 21 (24.7)	*n* = 85 (100)	
Gender *n*(%)	Male	10 (26.3)	13 (34.2)	3 (7.9)	1 (2.6)	11 (28.9)	38 (44.7)	0.072
	Female	12 (25.5)	8 (17.0)	13 (27.7)	4 (8.5)	10 (21.3)	47 (53.3)	
Age in years Md (IQR)		41.5 (33-46.8)	33 (29-38.5)	28.5 (22.3–38.3)	58 (49.5–61)	57 (49.5–66)	NA	< 0.001
Age *n*(%)	18–30	5 (21.7)	9 (39.1)	9 (39.1)	0 (0.0)	0 (0.0)	23 (27.7)	NA
	31–40	5 (22.7)	8 (38.1)	4 (25)	0 (0.0)	3 (15.8)	20 (24.1)	
	41–50	10 (45.5)	2 (9.5)	1 (6.2)	1 (6.2)	2 (10.5)	16 (19.3)	
	51–60	1 (4.5)	2 (9.5)	1 (6.2)	4 (80)	7 (36.8)	15 (18.1)	
	> 60	1 (4.5)	0 (0.0)	1 (6.2)	0 (0.0)	7 (36.8)	9 (10.8)	
CT Value Md (IQR)		NA	27.5 (22.8–33.2)	27.9 (21.7–35.8)	25.5 (22.6–33.9)	31.7 (26.3–36.2)	NA	0.45
HIV *n* (%)		6 (27.3)	3 (14.3)	1 (6.3)	0 (0.0)	1 (4.8)	11 (12.9)	0.154
Comorbidities *n*(%)	Hypertension	3 (13.6)	3 (14.3)	1 (6.3)	2 (40)	9 (43)	18 (52.9)	0.033
	Diabetes	0 (0.0)	1 (4.8)	0 (0.0)	0 (0.0)	6 (28.6)	7 (20.6)	0.003
	Asthma	1 (4.5)	1 (4.8)	0 (0.0)	0 (0.0)	2 (9.5)	4 (11.8)	0.713
	Gastritis	0 (0.0)	1 (4.8)	2 (12.5)	0 (0.0)	0 (0.0)	3 (8.8)	0.228
	Sinusitis	0 (0.0)	1 (4.8)	1 (6.3)	0 (0.0)	0 (0.0)	2 (5.9)	0.601

**Legends**: Not applicable (NA); Median (Md); Interquartile range (IQR); Cycle threshold (CT value), With an α = 0.05 * Kruskal-Wallis test

### SARS-CoV-2 infection is associated to a decrease on peripheral blood absolute counts of lymphocytes and eosinophils, below the reference values and no change on red blood series

**Fig. 1:**
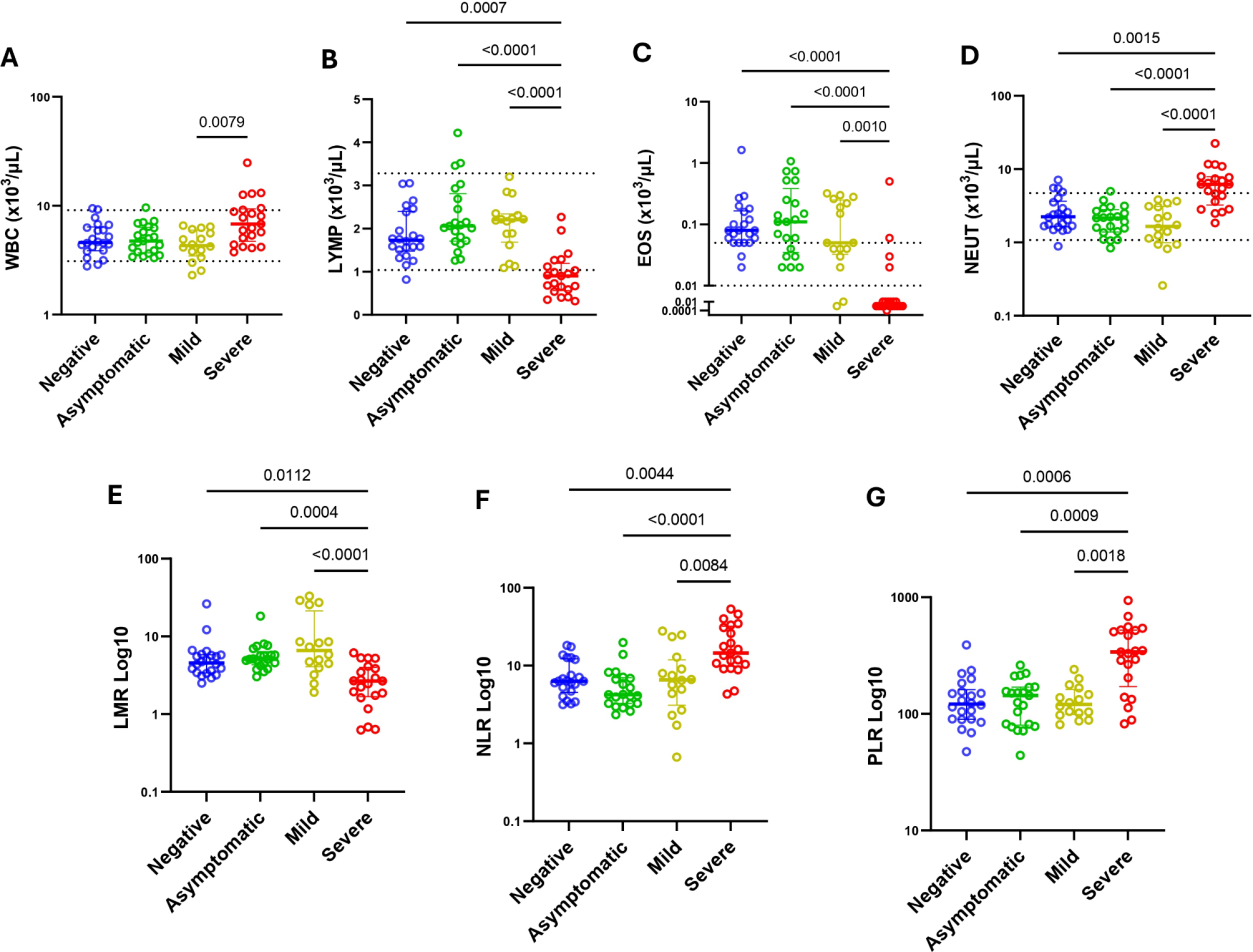
Associations of clinical state stratified into negative (*n* = 22), asymptomatic (*n* = 21), mild (*n* = 16), and severe (*n* = 21) cases with hematological parameters (**A**) White blood cells (WBC); (**B**) Lymphocytes (LYMP); (**C**) Eosinophils (EOS); (**D**) Neutrophils (NEUT); (**E**) Lymphocyte-to-Monocyte Ratio (LMR); (**F**) Neutrophil-to-Lymphocyte Ratio (NLR) and (G) Platelet-to-Lymphocyte Ratio (PLR) determined using the Kruskal‒Wallis test and adjusted by Dunn test with α = 0.05 Dashed line indicates parameter Mozambican reference value

**Fig. 2:**
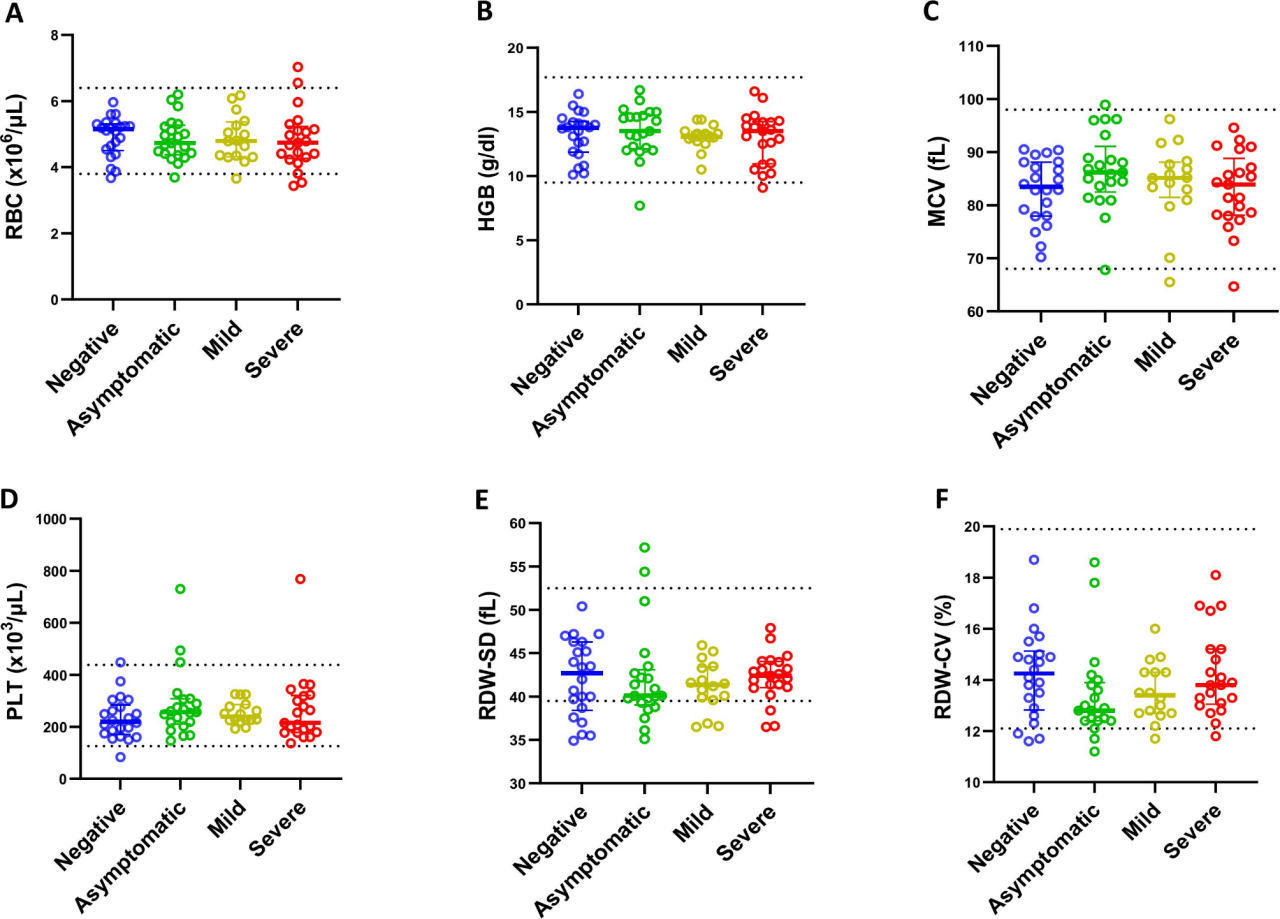
Association of clinical state stratified in negative, asymptomatic, mild, and severe groups, with hematological parameter (**A**) Red blood cells (RBC), (**B**) Haemoglobin (HGB), (**C**) Mean Corpuscular volume (MCV), (**D**) Platelet (PLT), (**E**) Red blood cell distribution width standard deviation (RDW-SD), (**F**) Red blood cell distribution width coefficient of variation RDW-CV, using Kruskal-Wallis Test and adjusted by Dunn Test with α = 0.05. Dashed line indicates parameter Mozambican reference value

Regarding haematological parameters, SARS-CoV-2 positive cases had a significantly (*p* = 0.0141) lower median EOS count (0.03 × 10^3^ cells/µL; IQR:0.01–0.15 × 10^3^ cells/µL) that did SARS-CoV-2 negative cases (0.08 × 10^3^ cells/µL; IQR:0.05–0.17 × 10^3^ cells/µL) (Additional file 3). Analysis of the cases, stratified by clinical presentation, revealed that severe cases presented significant higher counts of NEUT (*p* < 0.0001) PLR (*p* < 0.0001) and NLR (*p* < 0.0001), and significant lower counts of LYMP (*p* < 0.0001) and EOS (*p* < 0.0001) below the reference ranges and LMR (*p* < 0.0001), when compared to negative, asymptomatic, and mild cases. The median WBC count was significantly high (*p* = 0.007) only in the severe cases, compared to mild cases (Fig. 1). No significant (*p* > 0.05) changes were seen in the levels of RBC, HGB, RDW-SD, RDW-CV and PLT of p SARS- among the cases (Fig. 2).

Furthermore, we did not find significant differences in the levels of WBC, NEUT, LYMP and EOS, (*p* = 0.44; *p* = 0.68; *p* = 0.83 and *p* = 0.84 respectively) between males and females with SARS-CoV-2 infection. However, we saw a significant increase in NEUT concentration with age (*r* = 0.68; *p* < 0.0001) in female participants with COVID-19. This correlation was not seen in males.

### SARS-CoV-2 infection is associated to an increase on serum levels of C-reactive protein and glucose above the reference values

**Fig. 3:**
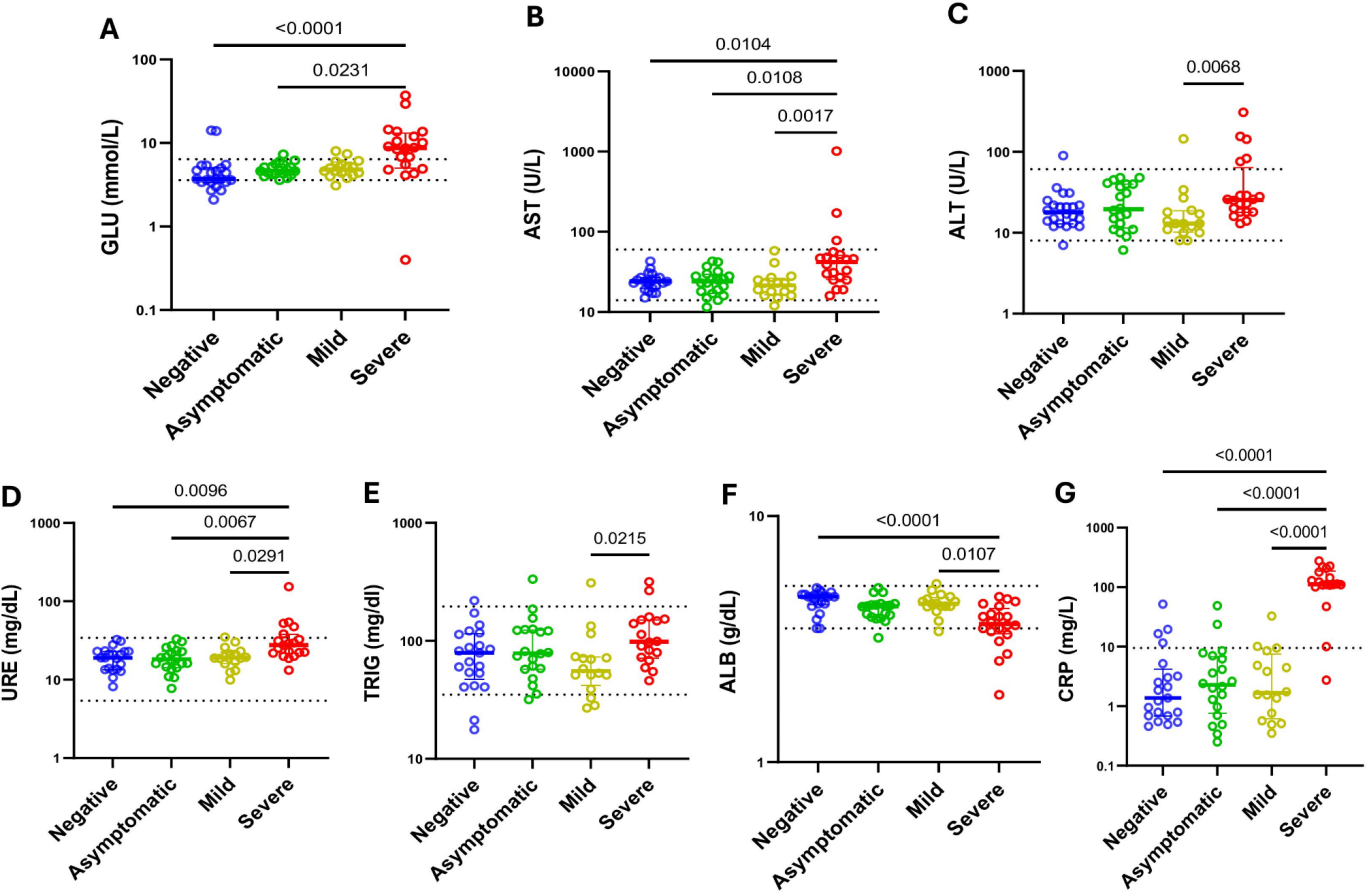
Association of clinical state stratified in negative (*n* = 22), asymptomatic (*n* = 21), mild (*n* = 16), and severe (*n* = 21) cases with biochemical parameter (**A**) Glucose, (GLUC); (**B**) Triglycerides, (TRIG); (**C**) Alanine transaminase, (AST); (**D**) Aspartate transaminase, (ALT); (**E**) Albumin, (ALB); (**F**) Urea, (URE); (**G**) C-reactive protein, (C-RP) using Kruskal‒Wallis Test and adjusted by Dunn Test with α = 0.05. Dashed line indicates parameter Mozambican reference value

Regarding the biochemical parameters, it was found significant higher median GLUC (5.1mmol/L; *p* = 0.0006), and C-RP (7.5 mg/L; *p* = 0.0068;) levels, and lower median ALB (4.2 g/dl; *p* = 0.0004;) levels on SARS-CoV-2 positive when compared to negative cases (3.7mmol/l; 1.4 mg/l; 4.7 g/dl respectively) see Additional file 4. Analysis of SARS-CoV-2 positives cases stratified by clinical presentation showed higher median AST (*p* = 0.0007), URE (*p* = 0.02), GLUC (*p* = 0.0001) and C-RP (*p* < 0.0001) levels in severe cases than in negative, asymptomatic, and mild cases (Fig. 2). GLUC and C-RP median levels on severe cases were above the reference ranges, having C-RP a 12-fold rise (Fig. 3). ALT and TRIG were significantly (*p* = 0.01 and *p* = 0.029 respectively) increased in severe cases than in mild cases, and ALB was the only parameters with significant low median levels on the severe cases (*p* < 0.001) when compared to mild and negatives cases (Fig. 3, Additional file 4). Comparison between sex, in those with SARS-CoV-2, showed significant median higher levels of ALT (26U/L, *p* < 0.0138), AST (28U/L, *p* = 0.0266) and URE (25 mg/dl, *p* = 0.0032) on male when compared to female (17U/L; 22U/L; 19U/L, respectively). In addition, C-RP was strongly correlated with clinical presentation (*p* < 0.0001; *r* = 0.67) and age (*p* < 0.0001; *r* = 0.71) while GLUC was strongly correlated with age (*p* < 0.001; *r* = 0.58). Stratification by sex showed a strong correlation between clinical presentation and URE in male cases (*p* = 0.002; *r* = 0.56).

### SARS-CoV-2 infection is associated to a reduction on serum levels of classical proinflammatory cytokines as IL-1β, IL-6, INF-γ

**Fig. 4:**
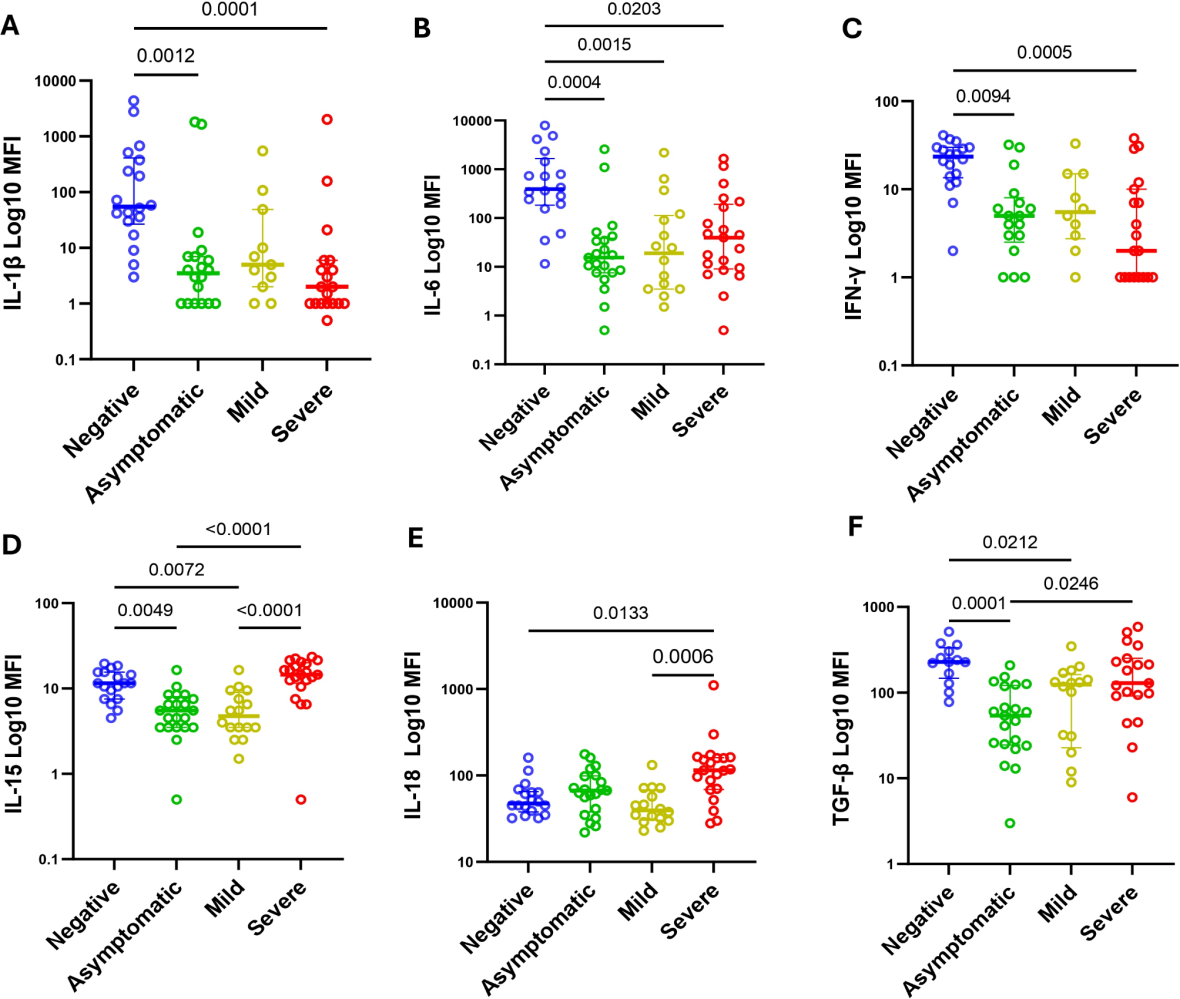
Association of clinical state stratified in negative (*n* = 22), asymptomatic (*n* = 21), mild (*n* = 16), and severe (*n* = 21) cases with cytokine (**A**) Interleukin-1 beta (IL-1β); (**B**) Interleukin-6, (IL-6); (**C**) Interferon Gama (IFN-γ); (**D**) Interleukin-15, (IL-15); (**E**) Interleukin-18, (IL-18) and (**F**) Transforming growth factor beta, (TGF-β) using Kruskal‒Wallis Test and adjusted by Dunn Test with α = 0.05

Compared to negative cases, SARS-CoV-2 positive cases, showed significant lower MFI levels of TNF-α (169 vs. 32; *p* < 0.0001), IL-6 (394 vs. 18; *p* < 0.0001), IL-8 (9698 vs. 995; *p* < 0.0001), IL-1β (55 vs. 2; *p* < 0.0001), IFN-γ (24 vs. 3; *p* < 0.0001), IL-4 (13 vs. 2; *p* < 0.0001), IL-5 (2 vs. 1; *p* = 0.008), IL-15 (12 vs. 7.5; *p* = 0.041), IL-17 (4.5 vs. 0.5; *p* < 0.0001), IL-21 (21 vs. 3; *p* < 0.0001), IL-2 (33 vs. 5; *p* < 0.0001) and TGF-β (229 vs. 102; *p* = 0.0005) see Additional file 5. Analysis of cytokine profiles stratified by clinical presentation, showed a significant decrease in IL-1β (*p* < 0.0001), IL-6 (*p* = 0.0203) and INF-γ (*p* = 0.0004) and increase in IL-18 (*p* = 0.0133) in severe cases when compared to negative cases (Fig. 3). Severe cases also showed significantly increase on IL-10 (*p* = 0.021) when compared to mild cases and IL-15 (*p* < 0.0001) and TGF-β (*p* = 0.024) when compared to asymptomatic (Fig. 4 and Additional file 6). The levels of all other tested cytokines were significantly (*p* < 0.05) lower in severe cases than in negative cases (Additional file 6). Analysis of the associations of these cytokines with a significant increase in severe cases, do not show differences when compared between sex (*p* > 0.05). When taken as a continuous variable, clinical presentation was correlated with IL-15 levels (*p* > 0.0001; *r* = 0.5).

### Haematological and biochemical signature distinguish severe COVID-19 cases and Cytokine signature distinguish negative and severe COVID-19 cases

**Fig. 5:**
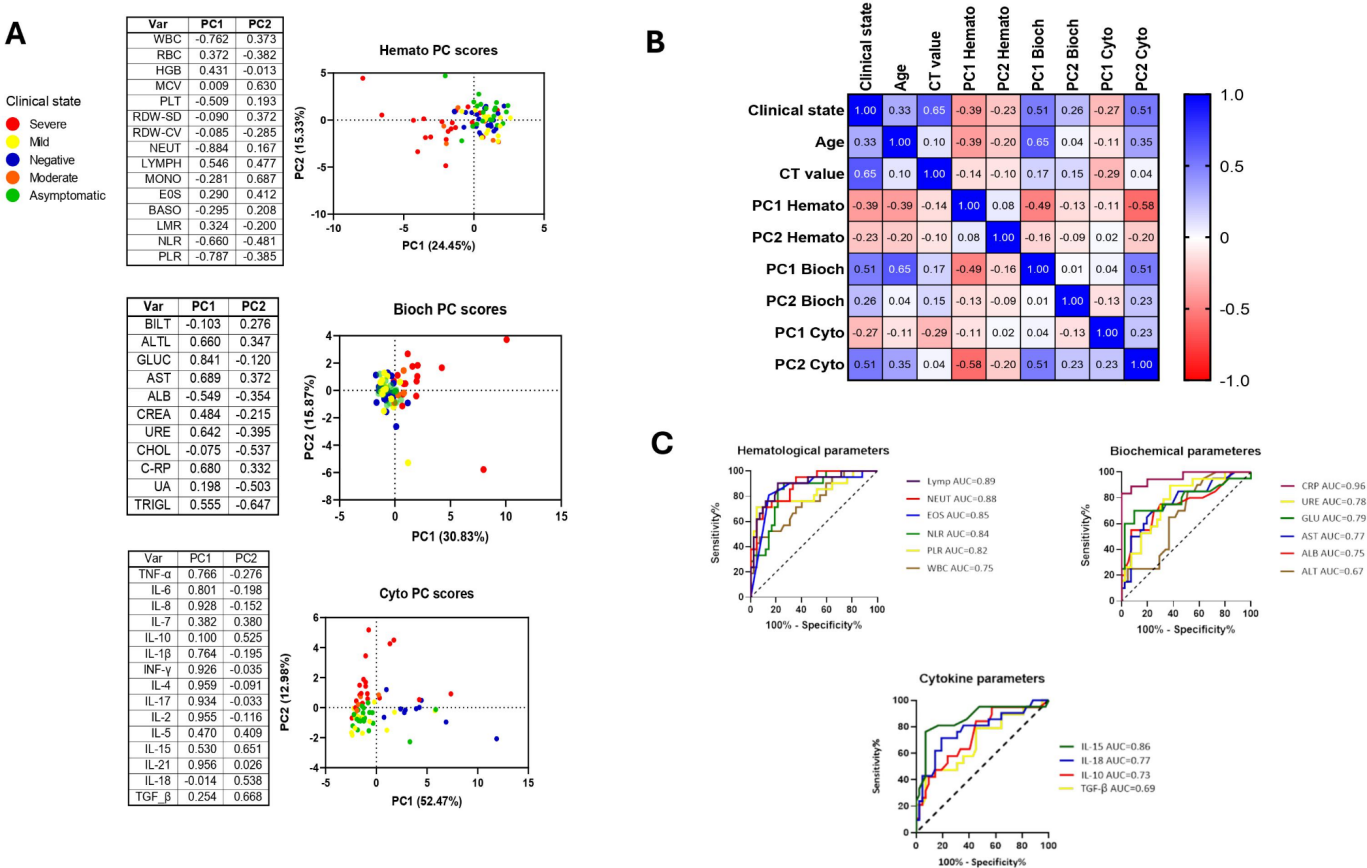
SARS-CoV-2 severity associated parameters from hematology, **biochemistry and cytokines looking at** (**A**) Principal Component (PC) analysis of grouped parameters stratified by clinical states negative (*n* = 22), asymptomatic (*n* = 21), mild (*n* = 16), and severe (*n* = 21) indicated by color; (**B**) Correlation matrix of clinical state (as a continuous variable not including negative cases), age (in years), CT value and PC scores from previously analysis using Spearman Rank with α = 0.05. Results are shown as r values, with the strength of correlation as follows: 0-0.19 (very weak), 0.2–0.39 (weak), 0.40–0.59 (moderate), 0.6–0.79 (strong) and 0.8-1 (very strong); (**C**) Receiver Operator Curves (ROC) of severity associated parameters classifying non-severe (*n* = 42) from severe (*n* = 21) cases. The results are shown as Area Under the Curve (AUC) with the interpretation as follows: 0.5–0.6 (failed), 0.6–0.7 (worthless), 0.7–0.8 (poor), 0.8–0.9 (good), > 0.9 (excellent), with a α = 0.05

We analyzed the distribution of the study population on principal component analysis (PCA) graphs by analyzing haematological, biochemical and cytokine data (Fig. 4A-C) clustering the cases and allowing the identification of parameters similar among the severe group. The pattern of severe cases was found to be associated to the first principal component (PC1) for both haematological and biochemical parameters and to be associated with the second principal component (PC2) for cytokine parameters (Fig. 5A). Additionally, characteristics of the negative group were only well distinguished on the cytokine PCA graph (Fig. 5A) and were associated with PC1. Based on the loadings of the PCs (close to each graph on Fig. 4A), the parameters with the largest effect on the haematological PC1 component were WBC, PLT, NEU, LYMP, NLR and PLR. The biochemical PC1s included ALT, GLUC, AST, ALB, URE, TRIG and C-RP and cytokine PC2s included IL-10, IL-15, IL-18 and TGF-β.

**Fig. 6:**
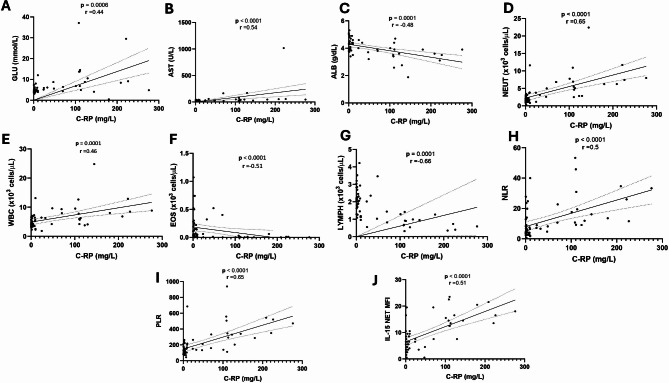
Moderate and strong correlation plots using positive rt-PCR SARS-CoV-2 of C-RP with (**A**) Glucose, (GLUC); (**B**) Alanine transaminase, (AST); (**C**) Albumin, (ALB); (**D**) Neutrophils, (NEUT); (**E**) White Blood Cells, (WBC); (**F**) Eosinophils, (EOS); (**G**) Lymphocyte, (LYMP); (**H**) Neutrophils to Lymphocyte Ratio, (NLR); (**I**) Platelets to Lymphocyte Ratio, (PLR) and (**J**) Interleukin-15 (IL-15) using Spearman rank with a α = 0.05. Results are shown as r values, with the strength of correlation as follows: 0-0.19 (very weak), 0.2–0.39 (weak), 0.40–0.59 (moderate), 0.6–0.79 (strong) and 0.8-1 (very strong)

When used as numerical data, clinical presentation was positively correlated with age (*p* = 0.004; *r* = 0.52) and CT value (*p* = 0.003; *r* = 0.53) without negative cases. Age was correlated with PC1 haematological (*p* < 0.001; *r*=-0.38), PC1 biochemical (*p* < 0.001; *r* = 0.65) and PC2 cytokines (*p* = 0.003; *r* = 0.35). PC2 cytokines were strongly correlated to PC1 haematological (*p* < 0.001; *r*=-0.57) and PC1 biochemical, (*p* < 0.001; *r* = 0.50) see Fig. 5B. We assessed the performance of parameters associated with severity, to classify non-severe and severe cases and results showed C-RP had an excellent performance with an AUC of 0.95 (CI:0.90-1.0; *p* < 0.001). Among the other parameters, LYMPH and IL-15 presented a good performance with an AUC of 0.88 (CI:0.80–0.98; *p* < 0.001) and 0.86 (CI:0.75–0.97; *p* < 0.001), respectively (Fig. 5C). When looking at the correlation of C-RP with other severity parameters in the haematological, biochemical, and cytokine profile, AST (*p* < 0.0001, *r* = 0.54), LYMPH (*p* = 0.0001, *r*=-0.66) and IL-15 (*p* < 0.0001, *r* = 0.51) respectively, had moderate to strong correlations within the cases (Fig. 6), and the other parameters showed weak correlations (see Additional file 7).

## Discussion

In this study we aimed to identify signatures of the SARS-CoV-2 infection in Mozambican patients based on haematological, biochemical, and cytokine profile, since diseases signatures allow the identification of useful tools which can be used on the distribution and correct allocation of services on sites with limited resources like Mozambique. In our cohort, we found that severe SARS-CoV-2 infection was characterized by a decrease in the haematological parameters EOS, LYMPH and LMR, and the biochemical parameters ALB, and to an increase in the haematological parameters WBC, NEUT, NLR and PLR, the biochemical parameters GLUC, ALT, AST, URE and C-RP and the cytokines parameters IL-15, IL-10, IL-18 and TGF-β. All of these parameters have already been associated with disease severity in previous reports [7, 8, 19, 20]. Unlike other publications, the peculiarities of this cohort were the lack of variation in the levels of the red series parameters and the downregulation of the production of classical proinflammatory cytokines such as IL-6 and TNF-α. Here we did not observe a significant variation in the red series or coagulation parameters as in a metanalysis with studies conducted in European and Asian countries reporting a strong association of both RBC counts and HGB concentrations with severity [13], and also a decrease in PLT numbers [12, 21]. Additionally, unlike in previous reports [7, 22–24], we did not observe an increase on the release of classical proinflammatory cytokines such as IL-1β, IL-6, IL-8, TNF-α or INF-γ. In the contrast, in our study, these cytokines were found to be present at lower levels in subjects with SARS-CoV-2 infection, especially in severe ones, than in negative cases. MFI levels of classic proinflammatory cytokines such as IL-6 and TNF-α were 21, 5 and 8-fold lower in SARS-CoV-2 positive cases than in negative cases.

Exacerbated PLT activation (coagulopathy) and RBC apoptosis have been found to be induced by an increase in IL-1β, IL-6 and IL-8 [25], whereas TNF-α inhibits the erythropoiesis cycle [26]. We hypothesize that the downregulation of these cytokines, observed in our group of severe cases explains the lack of variation in RBC and PLT counts. Furthermore, IL-6 and TNF-α have been associated with the onset of symptoms, length of hospital stay, respiratory failure and organ damage in many reports [27–29]. Previously, studies have shown that the African population produces lower levels of proinflammatory cytokines when stimulated, compared to population from Europe, America, and Asia [30]. A Ghanian study of COVID-19 patients also found low levels of IL-6 in the study cohort than in the European cohort in early phase of disease. As in our study, the levels were lower in the positive population than in the negative population [31].

We hypothesize that the SARS-CoV-2 virus induces a tolerable response in our cohort, explaining the low serum levels of cytokines in our study. Individuals who do not tolerate this stimulation, experience an increase in the production of cytokines, leading to hyperactivation a cytokine storm. In a tolerated response, due to constant stimulation with the same antigen, the innate response cells reduce their state of activation, which is reflected by expression of cytokines and other activation proteins [32]. Studies have pointed out to the possible role of *Plasmodium* sp., the causative agent of Malaria, a disease endemic in Mozambique, in inducing tolerance and is antigen similarity with SARS-CoV-2 [33]. However, more evidence not explored in this study is needed to clarify the role of the innate immune response on COVID-19 immunopathogenesis.

An increase on the IL-10 levels have been reported in many studies [7, 24, 34, 35], and some have proposed it as a target for immunotherapies. IL-18 was found to be elevated in severe cases and non survivors in other reports [36] as was IL-15 [37]. A study in the South African population [22] found IL-18 and IL-15 were associated with disease severity, as both were increased upon Intensive Care Unit (ICU) admission and were significantly lower in survivors than in non-survivors. In the same study, a significantly decrease in IL-10 and TGF-β levels was observed on day 7 of ICU admission of non-survivor patients. IL-15 and IL-18 participate in the maturation and activation NK cells, which are important for viral clearance [38, 39], for the studied population, innate immunity as plays a major role in the immune response.

Among all the studied markers, C-RP presented an excellent performance in discriminating non-severe from severe cases compared to performance shown by LYMPH (among the haematological parameters) and IL-15 (among the cytokines). C-RP have been used as a marker of many other lung diseases, and predictive values have already been proposed [40]. However, when looking at the potential for immunotherapies, IL-15 appears to be a suitable target, as shown in previous studies [41].

## Conclusions

In this study, COVID-19 inflammatory signature was found to be similar to the signature from previous study and to note a signature with alterations on several haematological and biochemical parameters accessible and not used on clinical contest such as the lymphocytes ratios. The study peculiarities were the unchanged on the red blood series and the decrease on blood levels of IL-6, TNF-α and IL-1β classical pro inflammatory cytokines. Our results suggest a major role of IL-15 in the inflammatory process. Studies like this highlight the importance of describing the peculiarities of a population to propose suitable markers based on evidence and not generalized from other studies from different settings. More studies are needed to validate these results for the Mozambican population, and those are the studied limitations, related to study design, which does not allow for a precisely estimate of the period during which these parameters can be accessed for patient evaluation and additionally the number of participants. A longitudinal design would have provided more information on the development of these parameters during infection. Additionally, different results might be related to how patient cases were categorized (severe and non-severe or survivor and non-survivor, etc.). Because the onset of symptoms was reported by patients, and many attend the hospital after trying homemade medicines or ignoring earlier sings, the exact phase of infection was not clear for all patients. Therefore, it is possible that the cohort does not reflect the acute phase of infection.

## Electronic supplementary material

Below is the link to the electronic supplementary material.

Supplementary Material 1

Supplementary Material 2

Supplementary Material 3

Supplementary Material 4

Supplementary Material 5

Supplementary Material 6

Supplementary Material 7

## Data Availability

The datasets used and/or analyzed during the current study are available from the corresponding author on reasonable request.

## Abbreviations

WBC: White blood cells
HGB: Haemoglobin
MCV: Mean Corpuscular volume
PLT: Platelets
RDW-SD: Red blood cell distribution width standard deviation
RDW-CV: Red blood cell distribution width coefficient of variation
NEUT: Neutrophils
LYMP: Lymphocytes
MONO: Monocytes
EOS: Eosinophils
BASO: Basophils
LMR: Lymphocyte-to-Monocyte ratio
NLR: Neutrophils-to-Lymphocyte Ratio
PLR: Platelets-to-Lymphocyte Ratio
AST: Aspartate aminotransferase
ALT: Alanine aminotransferase
ALB: Albumin
CREA: Creatinine
GLUC: Glucose
BILT: Total Bilirubin
URE: Urea
UA: Uric Acid
CHOL: Cholesterol
TRIG: Triglycerides
C-RP: C-reactive protein
IL: Interleukin
IL-1β: Interleukin 1 beta
INF-γ: Interferon gamma
TNF-α: Tumour Necrosis Factor alfa
TGF-β: Transform growth factor beta
