# Supplementary material for: COVID-19 inflammatory signature in a Mozambican cohort: unchanged red blood series and reduced levels of IL-6 and other proinflammatory cytokines
Source: BMC Infect Dis. 2024 Nov 11;24:1279. doi: 10.1186/s12879-024-10132-6 (PMC11555969; doi:10.1186/s12879-024-10132-6)
Supplement: Supplementary file 1 — Supplementary Material 1 [file 12879_2024_10132_MOESM1_ESM.pdf]

INSTITUTO NACIONAL DE SAÚDE MOÇAMBIQUE	Instituto Nacional de Saúde (INS)	CISPOC CENTRO DE INVESTIGAÇÃO E TREINO EM SAÚDE DA POLANA CANIÇO
Review: 0.1	Moringa-COVID-19 study data collection form	FM – CISPOC- CL225

A. Demographics

1.Name:__________________________2. Admission date: ___/___/___ 3. Department: _______________

4.Patient code:_________________________ Telephone Contact: _____________________________

5. Residence: Neighborhood_________________________________

6.Date of birth: ____/____/____ 7. Age: _________ 8. Sex: Male☐ Fem☐

9.Race: Black☐ White☐ Asian☐ Mixed race☐ Other: ___________________

10. Education: None☐ Primary☐ Secondary☐ Technical☐ University☐

11. Marital Status: Single☐ Married☐ Divorced☐ Widower☐

B. Epidemiological data and risk factors

12. Habits

a) Alcoholics ☐Duration _____ (days/months/years)

13. Recent trip: Yes☐ No☐ If yes locations: _________________________________

a) Departure date ___________________ b) Return date__________________

14. Contact with sick people: Yes☐ No☐ a) Duration of contact__________________

15.Presence of comorbidities

a)Diabetes☐ c) Asthma☐ e) Tumors☐

b) Hypertension☐ d) Heart diseases☐ f) Renal insufficiency☐

C. CLINICAL INFORMATION

16. Previous hospitalization: Yes☐ No☐
17. Health unit:___________________ 18. I**npatient service:** _______________________

19. Length of stay:______________

20. Use of medical devices: Yes☐ No☐

a) Venous catheter: Yes☐No☐

b) Mechanical ventilation: Yes☐No☐

c) Nasogastric tube: Yes☐No☐

d) Surgical procedures: Yes☐No☐

e) Others:____

f) **Vaccination:**Yes☐ No☐ which: ____

21. Medication: prior treatment: Yes☐ No☐

Name **Duration** **Start Date**

___________________________ Duration ___days ___ ___ ___ dd/mm/yy

___________________________ Duration ___days ___ ___ ___ dd/mm/yy

___________________________ Duration ___days ___ ___ ___ dd/mm/yy

D. Clinical data

22.Transfer: Yes☐ No☐ **23. Health unit:** _______________ **24. Service**: _______________

25. Symptom onset date: ___/___/___ **26. Date of admission:**___/___/___

27. Days of symptom evolution:________

28. Inpatient diagnosis:____

____

29. Symptomatology

Fever☐ Myalgia☐ Diarrhea☐ Splenomegaly☐ Cough☐
Chills☐ Anorexia☐ Vomiting☐ Hepatomegaly☐ Chest pain☐
Headache☐ Dehydration☐ Abdominal pain☐ Oropharyngeal pain☐ Dyspnea☐
Athralgia☐ Nausea☐ Jaundice☐ Spine pain☐
Weakness☐ Pain when urinating☐

Others:____

30. Serological status (HIV): Yes☐ No☐ **31. CD4 level** ________ **32. Viral load**________

33. Hematological Data:

Parameter	Result	Reference values<br>Men Both sexes Women
HGB		12.2 - 17.7 9.5 - 15.8
PLT		126-438
WBC		3.1-9.1
Neutrophils		1.08 - 4.71
Lymphocytes		

Investigator's Signature:_____________________________________ Date:_____/_____/_____

Verified by:____ Date: _____/_____/_____
