# Supplementary material for: COVID-19 inflammatory signature in a Mozambican cohort: unchanged red blood series and reduced levels of IL-6 and other proinflammatory cytokines
Source: BMC Infect Dis. 2024 Nov 11;24:1279. doi: 10.1186/s12879-024-10132-6 (PMC11555969; doi:10.1186/s12879-024-10132-6)
Supplement: Supplementary file 2 — Supplementary Material 2 [file 12879_2024_10132_MOESM2_ESM.pdf]

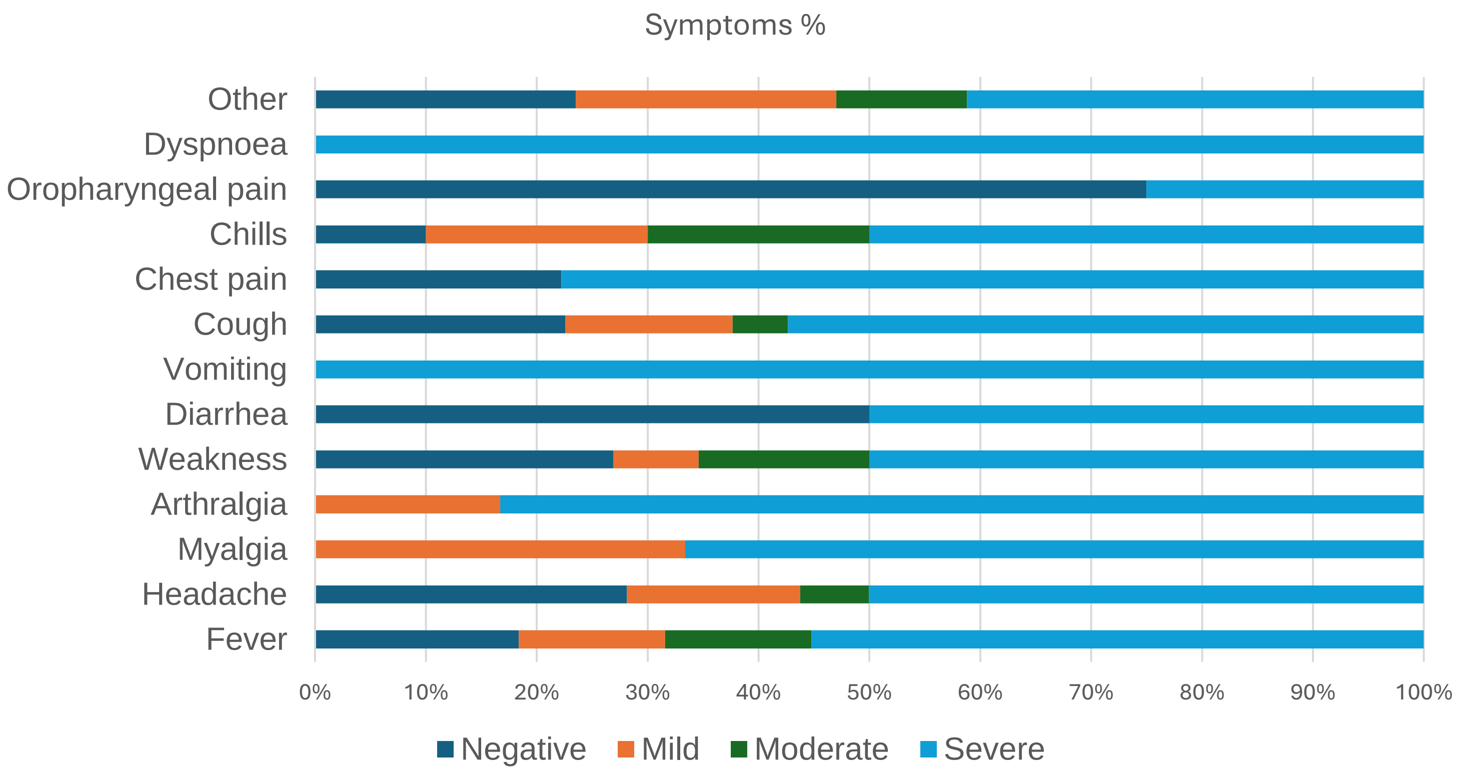

Figure S1 related to Table 1. Symptoms frequency stratified by clinical state (negative, mild, moderate and severe).
