# Supplementary material for: COVID-19 inflammatory signature in a Mozambican cohort: unchanged red blood series and reduced levels of IL-6 and other proinflammatory cytokines
Source: BMC Infect Dis. 2024 Nov 11;24:1279. doi: 10.1186/s12879-024-10132-6 (PMC11555969; doi:10.1186/s12879-024-10132-6)
Supplement: Supplementary file 3 — Supplementary Material 3 [file 12879_2024_10132_MOESM3_ESM.pdf]

Table S1 related to Figure1 and Figure 2. Median level of haematological parameters according to SARS-CoV-2 infection severity

Parameters	Reference values	SARS-CoV-2 PCR result			SARS-CoV-2 positive cases clinical presentation			
		Negative	Positive	P value*	Asymptomatic	Mild	Severe	P value**
		Md (IQR)	Md (IQR)		Md (IQR)	Md (IQR)	Md (IQR)	
**WBC(x10³/µL)**	3.1-9.1	4.6 (3.9-6.4)	5.2 (3.9-6.9)	0.5057	4.74 (3.7-6.325)	4.315 (3.463-5.958)	6.8 (4.725-9.34)	0.0065
**RBC (x10⁶/µL)**	3.8-6,4	5.2 (4.5-5.3)	4.8 (4.4-5.3)	0.3267	4.73 (4.385-5.27)	4.795 (4.32-5.365)	4.74 (4.265-5.225)	0.7721
**HGB (g/dl)**	9.5-17,7	14 (12-14)	13 (12-14)	0.7707	13.5 (12.1-14.85)	13.05 (12.75-13.45)	13.5 (10.95-14.25)	0.5522
**MCV (fL)**	68-98	83 (78-88)	85 (81-88)	0.3316	86.2 (82.5-91.05)	85.15 (81.5-88.15)	83.9 (78.1-88.85)	0.272
**PLT (x10³/µL)**	126- 438	220 (171-283)	249 (202-306)	0.1589	257 (210-307)	239.5 (225.5-284.8)	215 (178.5-320.5)	0.3999
**RDW-SD (fL)**	39.5- 52.5	43 (38-46)	42 (40-44)	0.652	12.8 (12.4-13.9)	13.4 (12.7-14.3)	13.8 (13.05-15.2)	0.0839
**RDW-CV (%)**	12.1-19.9	14 (13-15)	14 (13-14)	0.2233	13.2 (11.9-14.7)	12 (10.4-15)	12.3 (11-13.8)	0.37
**NEUT (x10³/µL)**	1.08- 4.71	2.3 (1.7-3.7)	2.7 (1.6-5)	0.5089	2.16 (1.395-2.81)	1.655 (0.99-3.103)	6.22 (3.24-7.865)	<0.0001
**LYMPH (x10³/µL)**	1.04-3.28	1.7 (1.5-2.4)	1.6 (0.96-2.2)	0.2796	2.06 (1.71-2.81)	2.205 (1.685-2.34)	0.9 (0.565-1.195)	<0.0001
**MONO (x10³/µL)**	0.21-0.88	0.41 (0.32-0.46)	0.39 (0.26-0.49)	0.8479	0.42 (0.365-0.53)	0.385 (0.1375-0.4775)	0.31 (0.24-0.505)	0.4545
**EOS (x103/µL)**	0.01- 0.05	0.08 (0.05-0.17)	0.03 (0.01-0.15)	0.0141	0.11 (0.035-0.385)	0.05 (0.0325-0.25)	0 (0-0.01)	<0.0001
**BASO (x103/µL)**	0.01-1.07	0.02 (0.01-0.03)	0.01 (0.01-0.02)	0.1179	0.02 (0.01-0.025)	0.01 (0.01-0.02)	0.01 (0.005-0.03)	0.084
**LMR**	NA	4.6 (3.4-5.8)	4.5 (2.7-5.8)	0.4384	5.095 (4.33-6.377)	6.575 (4.081-21.36)	2.667 (1.682-3.994)	<0.0001
**NLR**	NA	6.3 (4.5-12)	8.2 (4.2-16)	0.3354	4.231 (3.258-6.993)	6.565 (3.105-11.87)	14.47 (9.797-32.06)	<0.0001
**PLR**	NA	121 (90-161)	156 (104-287)	0.0406	143.9 (79.78-169.4)	120.6 (98.52-161.8)	340 (171.4-522.2)	<0.0001

Legends: Not applicable (NA); Median (Md); Interquartile range (IQR); White blood cells (WBC), Hemoglobin (HGB), Mean Corpuscular volume (MCV), Platelet (PLT), Red blood cell distribution width standard deviation (RDW-SD), Red blood cell distribution width coefficient of variation RDW-CV, Neutrophil (NEUT), Lymphocyte (LYMP), Monocyte (MONO), Eosinophil (EOS), Basophil (BASO), Lymphocyte-to-Monocyte ratio (LMR), Neutrophils-to-Lymphocyte Ratio (NLR) and Platelets-to-Lymphocyte Ratio (PLR). With α=0.05 *Mann Whitney, **Kruskal-Wallis test
