# Supplementary material for: COVID-19 inflammatory signature in a Mozambican cohort: unchanged red blood series and reduced levels of IL-6 and other proinflammatory cytokines
Source: BMC Infect Dis. 2024 Nov 11;24:1279. doi: 10.1186/s12879-024-10132-6 (PMC11555969; doi:10.1186/s12879-024-10132-6)
Supplement: Supplementary file 4 — Supplementary Material 4 [file 12879_2024_10132_MOESM4_ESM.pdf]

Table S2 related to Figure 3. Median level of biochemical parameters according to disease severity of COVID-19

Parameters	Reference values	SARS-CoV-2 PCR Result			SARS-CoV-2 positive cases clinical presentation			P value**
		Negative Md(IQR)	Positive Md (IQR)	P value*	Asymptomatic M (IQR)	Mild M (IQR)	Severe M (IQR)	
GLUC (mmol/L)	3.6- 6.4	3.7 (3.4-5.1)	5.1 (4.3-7.4)	0.0006	4.65 (4.225-5.5)	4.8 (4.175-5.9)	8.75 (5.05-13.28)	0.0001
CHOL (mg/dL)	77- 208	126 (107-156)	134 (112-155)	0.7883	140.8 (124.1-179.8)	134.4 (104.3-151.4)	124.5 (93.2-144.6)	0.1563
TRIGL (mg/dL)	35- 195	79 (47-115)	74 (55-123)	0.5891	78.77 (57.75-124.3)	55.55 (42.04-73.01)	98.24 (71.46-151.1)	0.029
BILT (umol/L)	2.9- 37.0	6.8 (3.9-8.8)	5.1 (3-7.3)	0.1957	6.16 (4.455-10.09)	4.67 (2.223-7.925)	4.92 (2.763-7.15)	0.2786
AST (U/L)	14- 60	24 (20-27)	26 (19-42)	0.2238	24 (17.25-29.5)	21.5 (16.5-26.5)	42 (25.5-51)	0.0007
ALT (U/L)	8.0- 61	18 (13-23)	20 (13-36)	0.303	40 (19.5-48)	18.75 (13-145)	64.25 (25.5-309)	0.0109
C-RP (mg/L)	≤9.5	1.4 (0.69-4.2)	7.5 (1.6-103)	0.0068	2.275 (0.7675-6.858)	1.66 (0.6175-7.635)	112.2 (106.4-187.4)	<0.0001
ALB (g/dL)	3.5- 5.2	4.7 (4.4-4.8)	4.2 (3.7-4.4)	0.0004	4.3 (3.89-4.4)	4.4 (4.293-4.675)	3.65 (3.325-4.225)	<0.0001
CREA (mg/dL)	0.37- 1.1	0.9 (0.69-1)	0.75 (0.66-0.98)	0.3901	0.765 (0.6425-0.8875)	0.695 (0.6-0.795)	0.85 (0.7-1.13)	0.0779
UA (mg/dL)	1.53- 6.71	4.7 (3.8-5.6)	4.2 (3.5-5.5)	0.563	4.25 (3.275-5.5)	3.95 (3-5.475)	4.7 (3.5-6)	0.654
URE (mg/dL)	5.40- 34.23	19 (14-23)	21 (16-29)	0.2143	18.27 (14.26-23.33)	19.11 (16.26-22.66)	27.55 (21.77-38.35)	0.0024

Legends: Not applicable (NA); Median (Md); Interquartile range (IQR); Glucose, (GLUC); Cholesterol, (CHOL); Triglycerides, (TRIG); Total Bilirubin (BILT); Aspartate aminotransferase, (AST); Alanine aminotransferase, (ALT); C-reactive protein, (C-RP); Albumin, (ALB), Creatinine, (CREA); Uric Acid (UA); Urea, (URE). With an $\alpha=0.05$ *Mann Whitney, **Kruskal-Wallis test
