# Supplementary material for: COVID-19 inflammatory signature in a Mozambican cohort: unchanged red blood series and reduced levels of IL-6 and other proinflammatory cytokines
Source: BMC Infect Dis. 2024 Nov 11;24:1279. doi: 10.1186/s12879-024-10132-6 (PMC11555969; doi:10.1186/s12879-024-10132-6)
Supplement: Supplementary file 5 — Supplementary Material 5 [file 12879_2024_10132_MOESM5_ESM.pdf]

Table S3 related to Figure 4. Median level of cytokine parameters according to disease severity of COVID-19

Parameters	SARS-CoV-2 PCR result			SARS-CoV-2 positive cases clinical presentation			
	Negative Md (IQR)	Positive Md (IQR)	P-value*	Asymptomatic Md (IQR)	Mild Md (IQR)	Severe Md (IQR)	P-value**
IL-1β	55 (27-412)	2 (1-6)	<0.0001	3.5 (1-7)	5 (2-49)	2 (1-6)	<0.0001
IL-6	394 (184-1658)	18 (6.5-70)	<0.0001	15.5 (7.5-38.5)	19 (3.5-113)	39.5 (9-191)	<0.0001
TNF-α	169 (90-245)	32 (13-102)	<0.0001	54.5 (27-106.5)	53.75 (10.75-211.3)	16.5 (12-49.5)	0.0003
INF-γ	24 (14-30)	3 (1-7)	<0.0001	5 (2.5-8)	5.5 (2.75-15)	2 (1-10)	0.0004
IL-2	33 (24-48)	5 (2-10)	<0.0001	7 (3-12.75)	5.25 (1.25-13.75)	3 (2-8)	<0.0001
IL-4	13 (7-16)	2 (0.5-5)	<0.0001	3 (2-5)	2.5 (1-6.5)	2 (1-7)	0.0001
IL-5	2 (1-2)	1 (0-1)	0.0088	1 (1-2)	1 (0-1)	1 (0-1)	0.0276
IL-7	22 (19-26)	17 (10-31)	0.0888	16.75 (11.25-20.75)	13 (9.25-21)	32 (15.5-41)	0.008
IL-8	9698 (5376-13030)	995 (119-2371)	<0.0001	1640 (710-3459)	726.5 (69.75-4833)	213.5 (95-1296)	<0.0001
IL-15	12 (7.5-16)	7.5 (4-14)	0.0417	5.5 (3.5-8)	4.75 (3.5-9)	14.5 (11.5-20)	<0.0001
IL-17	4.5 (1.5-6.8)	0.5 (0-1.5)	<0.0001	0.5 (0.25-2)	0.5 (0-1.5)	0.5 (0.25-2)	0.0006
IL-21	21 (13-25)	3 (0-7.5)	<0.0001	5 (2.75-9)	5 (3-10.75)	6.5 (3-13)	0.0001
IL-18	48 (38-65)	68 (35-118)	0.1882	67 (38-99)	39.5 (30.5-68.25)	115 (69-161)	0.0006
IL-10	6.5 (6.5-11)	8.5 (2.5-18)	0.7887	8 (2.5-16.75)	4 (1.75-13.25)	16.5 (7.5-31.5)	0.0224
TGF-β	229 (148-334)	102 (32-176)	0.0005	54 (24.5-121.5)	124 (22.75-163)	129 (91.5-252)	0.0001

Legends: Median (Md); Interquartile range (IQR); Interleukin, (IL); Tumor Necrosis Factor, (TNF-α); Interferon gama, (INF-γ); (Transforming growth factor beta, (TGF-β); Total Bilirubin (BILT); With an α=0.05 *Mann Whitney, **Kruskal-Wallis test
