# Supplementary material for: COVID-19 inflammatory signature in a Mozambican cohort: unchanged red blood series and reduced levels of IL-6 and other proinflammatory cytokines
Source: BMC Infect Dis. 2024 Nov 11;24:1279. doi: 10.1186/s12879-024-10132-6 (PMC11555969; doi:10.1186/s12879-024-10132-6)
Supplement: Supplementary file 6 — Supplementary Material 6 [file 12879_2024_10132_MOESM6_ESM.pdf]

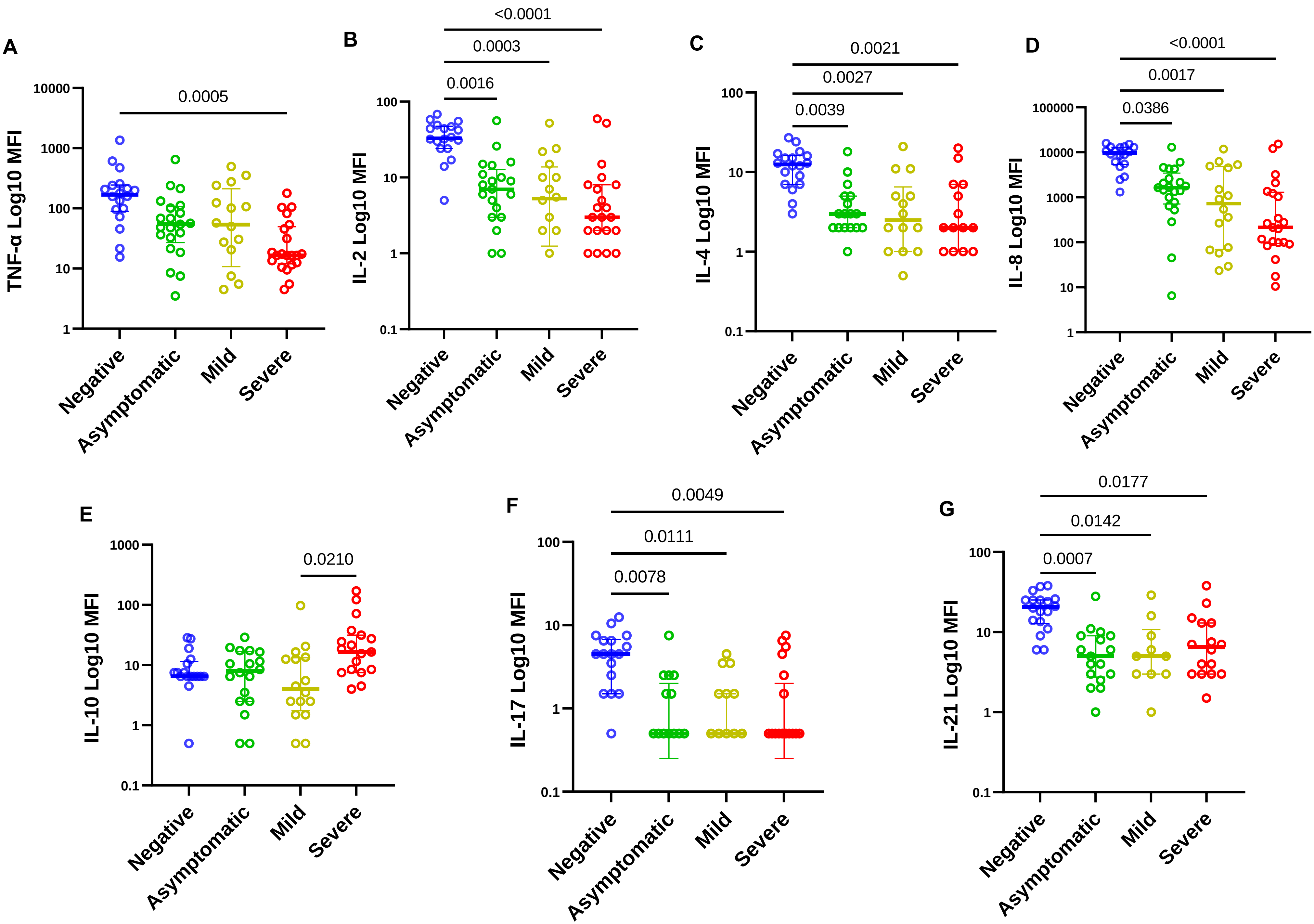

Figure S2 related to Figure 4. Association of clinical state stratified in negative (n=22), asymptomatic (n=21), mild (n=16), and severe (n=21) with cytokine **A.**Tumor necrosis factor alfa (TNF-α); **B.** Interleukin-2 (IL-2) **C.** Interleukin-4 (IL-4), **D.** Interleukin-8 (IL-8), **E.** Interleukin-10 (IL-10), **F.** Interleukin-17 (IL-17) and **G.** Interleukin-21 (IL-21) using Kruskal-Wallis Test and adjusted by Dunn Test with α=0.05
