# Supplementary material for: COVID-19 inflammatory signature in a Mozambican cohort: unchanged red blood series and reduced levels of IL-6 and other proinflammatory cytokines
Source: BMC Infect Dis. 2024 Nov 11;24:1279. doi: 10.1186/s12879-024-10132-6 (PMC11555969; doi:10.1186/s12879-024-10132-6)
Supplement: Supplementary file 7 — Supplementary Material 7 [file 12879_2024_10132_MOESM7_ESM.pdf]

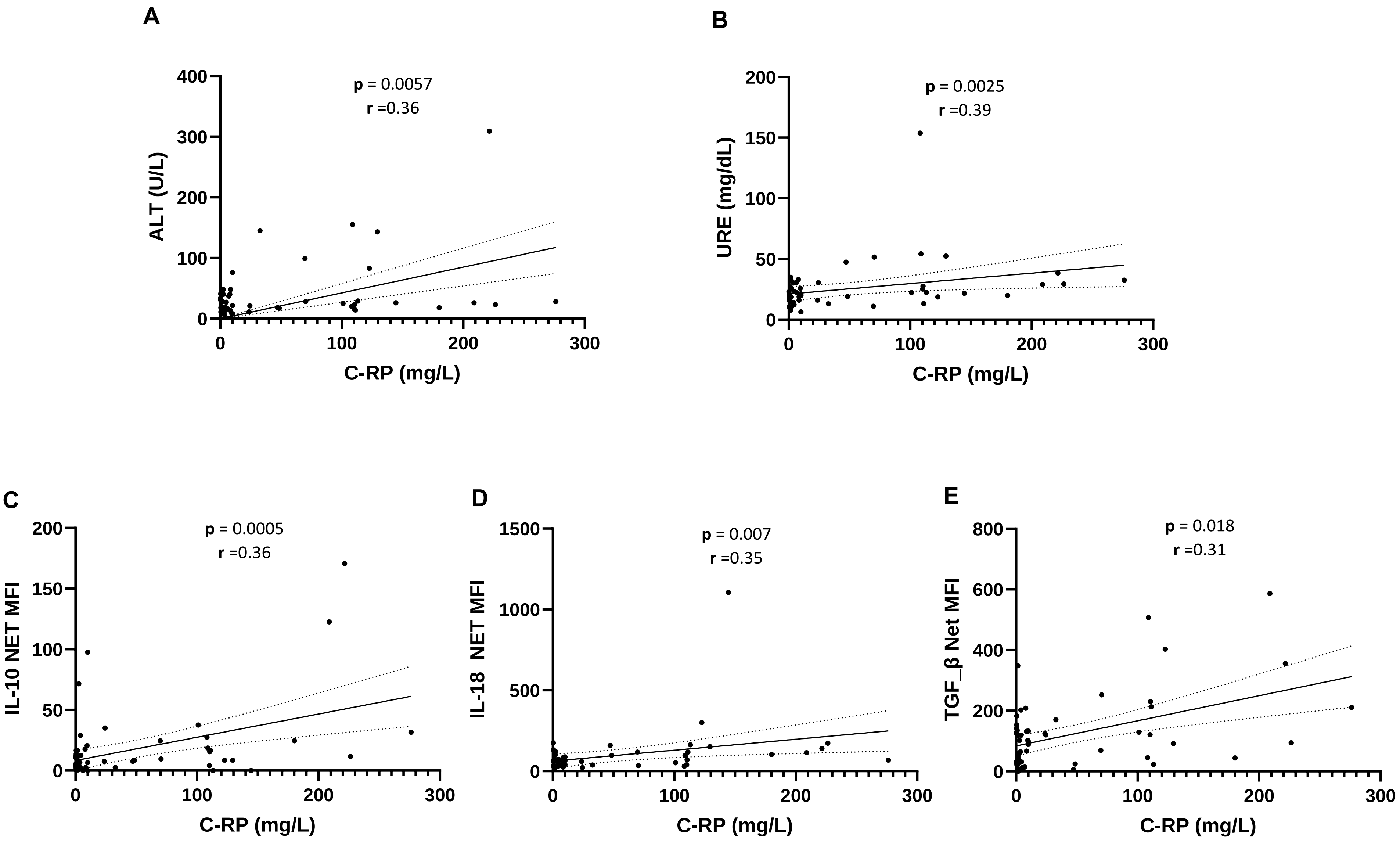

Figure S3 related to Figure 6. Weak Correlation plots of C Reactive Protein (C-RP) with **A.** Aspartate transaminase (AST), **B.** UREA (URE), **C.** Interleukin-10 (IL-10), **D.** Interleukin-18 (IL-18), **E.** Transforming growth factor beta (TGF-β) using Spearman rank with a α=0.05 Results are shown as r values, with the strength of correlation as follows: 0-0.19 (very weak), 0.2-0.39 (weak), 0.40-0.59 (moderate), 0.6-0.79 (strong) and 0.8-1 (very strong).
